# The spatial dynamics of urban vegetation and housing prices: Insights from pre- and post-pandemic Chicago using OLS and MGWR models

**DOI:** 10.1371/journal.pone.0330932

**Published:** 2025-09-05

**Authors:** Dasom Han, Chang Gyu Choi

**Affiliations:** Graduate School of Urban Studies, Hanyang University, Seoul, Republic of Korea; Gebze Teknik Universitesi, TÜRKIYE

## Abstract

This study examines the spatial dynamics of urban vegetation and its impact on housing prices in Chicago, analyzing data from both pre- and post-COVID-19 periods. Employing Ordinary Least Squares (OLS) and Multiscale Geographically Weighted Regression (MGWR) models, we assess how the effects of green spaces on property values vary across different neighborhoods. The OLS model generally indicates a positive correlation between increased vegetation and housing prices. In contrast, the MGWR model reveals that the benefits of urban green spaces to property values are not uniformly distributed and exhibit significant variability. Notably, in some South Side areas of Chicago, increases in green space correlate with declines in property values, a sensitivity that intensified post-pandemic, leading to notable price declines. Conversely, the North Side, characterized as a higher-income area, shows greater resilience to the impacts of both increased green spaces and the COVID-19 pandemic, with less susceptibility to economic downturns. This research underscores the intricate interplay between urban green spaces and economic factors, highlighting how local socio-economic conditions and urban planning strategies can influence the economic benefits of vegetation. The findings provide essential insights for urban policymakers and planners striving to promote sustainable development and equitable economic growth in urban environments.

## 1. Introduction

Urban vegetation is increasingly recognized as a critical component of sustainable city planning, contributing to both environmental quality and economic value. Green spaces have been shown to improve air quality, enhance the aesthetic appeal of neighborhoods, and offer recreational benefits, all of which can influence housing prices [1–2]. However, the dynamics between urban vegetation and property values are complex, particularly in the context of the COVID-19 pandemic, which has shifted both urban development patterns and housing market preferences.

This study aims to explore the evolving relationship between urban vegetation and housing prices before and after the pandemic. Previous research has consistently found a positive correlation between green spaces and property values, yet the spatial variability of these effects suggests that not all areas experience the same benefits. Factors such as local socioeconomic conditions and urban development trends can mediate the impact of green spaces, sometimes leading to unexpected decreases in property values.

The COVID-19 pandemic has further complicated this relationship. As remote work became more prevalent and people sought outdoor spaces to mitigate the effects of lockdowns, the demand for homes near green spaces surged in many urban areas. This research investigates how these changes have reshaped the housing market and what implications they hold for future urban planning.

By employing advanced spatial analysis techniques—Ordinary Least Squares (OLS) and Multiscale Geographically Weighted Regression (MGWR)—this study provides a detailed understanding of how changes in urban vegetation affect housing prices across different regions. Our findings offer valuable insights for policymakers and urban planners aiming to develop resilient and sustainable cities in a post-pandemic world.

## 2. Literature review

Urban green spaces have been consistently linked to increased property values, as they provide environmental benefits, recreational opportunities, and enhance mental well-being. Foundational research by Anderson and Cordell (1988) demonstrated that trees and green spaces in residential areas could increase property values by 3.5%–4.5%, emphasizing the long-term benefits of urban greenery [1]. Tyrväinen and Miettinen (2000) extended this line of research by investigating the effects of forest proximity on property values in Finland. Their study found that properties located near urban forests enjoyed a significant increase in value. This research highlighted the importance of different types of green spaces, including forests and parks, in urban planning and their varying impacts on housing prices [2]. Wolch et al. (2014) expanded on these findings by discussing how green spaces are unevenly distributed, often benefiting wealthier communities and potentially leading to gentrification in lower-income areas [3].

Panduro and Veie (2013) took a more detailed approach by categorizing green spaces based on their characteristics and location. They showed that well-maintained parks positively impacted property values, while green buffer zones near industrial areas often reduced property values. Their research also demonstrated a diminishing effect of proximity to green spaces after a certain distance [4].

The COVID-19 pandemic has further emphasized the value of green spaces. Malik, Kim, and Cultice (2023) explored how the rise of remote work during the pandemic increased the demand for private yard spaces, particularly in metropolitan areas like Chicago and New York, while the value of proximity to public parks remained relatively stable [5]. Similarly, Noszczyk et al. (2022) found that public appreciation for urban green spaces surged during the pandemic, as these spaces became vital for maintaining mental and physical health during lockdowns [6]. Geng et al. (2021) provided a global analysis of park visitation, showing that park usage increased significantly during the pandemic despite mobility restrictions [7].

Lai et al. (2020) highlighted how urban green spaces played a crucial role in mental health recovery during the pandemic. They found that access to these spaces helped alleviate stress and anxiety caused by social isolation, particularly in densely populated cities where outdoor spaces were limited [8]. Ugolini et al. (2020) similarly noted that during the pandemic, people increasingly turned to smaller local green spaces, such as neighborhood parks and gardens, instead of larger public parks, due to restrictions on movement [9].

Johnson et al. (2021) explored the relationship between COVID-19 transmission rates and park usage in the UK, finding that parks provided a safer alternative to indoor spaces for exercise and socializing, helping reduce transmission risks [10]. Li and Zhang (2021) analyzed housing price changes across various U.S. regions, revealing that suburban areas generally saw an increase in housing values, possibly influenced by a growing preference for residences with more space and access to natural environments, compared to more densely populated urban areas [11].

Xiao and Xu (2024) discussed how the COVID-19 pandemic necessitated significant adaptational strategies within the maritime transport sector, much like those required in urban planning. This sector faced logistical challenges and disruptions but showcased remarkable resilience, paralleling the adaptive measures needed for sustainable urban development [12]. Huang and Kwan (2023) examined the association between COVID-19 risk and various environmental and housing conditions in Hong Kong, using GPS-based data. Their study found that access to green spaces helped reduce the risk of COVID-19 transmission. Additionally, the research highlighted that poor housing conditions, such as overcrowding and inadequate maintenance, were associated with an increased risk of COVID-19, emphasizing the importance of considering housing quality in public health responses [13]. Li et al. (2022) further examined the impact of mobility restrictions on the rental housing market in large Chinese cities. Their research revealed that these restrictions led to short-term decreases in rental transactions and higher vacancy rates, with long-term effects still emerging [14].

Despite this growing body of research, there remains a gap in understanding the localized, spatial variability of these effects, particularly in post-pandemic contexts. Advanced spatial econometric techniques, such as Multiscale Geographically Weighted Regression (MGWR), have been underutilized in exploring how urban green spaces affect housing prices across different regions. This study aims to fill that gap by employing these advanced methods to analyze the spatial heterogeneity of urban vegetation's impact on housing prices, especially in the wake of the COVID-19 pandemic. By doing so, it offers insights that are crucial for policymakers and urban planners looking to foster sustainable urban development and enhance public health through green spaces.

## 3. Empirical strategy

This study aims to evaluate the impact of changes in green space accessibility on the capitalization effect of housing prices, particularly across the periods before and after the COVID-19 outbreak. We integrate a multi-level analysis framework that employs Ordinary Least Squares (OLS), Moran’s I test, and Multiscale Geographically Weighted Regression (MGWR) to provide insights into the spatial variability and scalability of these effects. ArcGIS Pro was utilized to process and analyze the spatial data, ensuring high precision in mapping and spatial analysis.

### 3.1 Ordinary Least Squares (OLS) analysis

The OLS approach facilitates a foundational understanding of the relationship between housing prices and proximity to green spaces while controlling for confounding variables such as characteristics of the property and the neighborhood. The model equation is:


$$P=\beta_{\text{0}}+\beta_{\text{1}}G+\beta_{\text{2}}C+\beta_{\text{3}}H+\beta_{\text{4}}N+\in$$

Where:

*P* denotes the housing price,

*G* represents the change in vegetation,

C indicates the period before and after the COVID-19 outbreak,

*H* represents individual housing characteristics,

*N* denotes variables of the surrounding environment,

ε is the error term.

### 3.2 Moran’s I test

As the Ordinary Least Squares (OLS) model does not inherently account for spatial autocorrelation, the initial analytical step involves employing the Global Moran’s I to assess the spatial correlation of housing prices in Chicago. This adjustment is crucial to set the stage for the more sophisticated Multiscale Geographically Weighted Regression (MGWR) model. The formula for Global Moran’s I is:


$$I=\frac{n}{\Sigma_{i=\text{1}}^{n}\Sigma_{j=\text{1}}^{n}w_{ij}}*\frac{\Sigma_{i=\text{1}}^{n}\Sigma_{j=\text{1}}^{n}w_{ij}(x_{i}-\overset{―}{x})(x_{j}-\overset{―}{x})}{\sum_{i=\text{1}}^{n}{\left(x_{j}-\overset{―}{x}\right)}^{\text{2}}}$$

Where:

n is the total number of observations (spatial objects),

$x_{i}$ and $x_{j}$ are the attribute values of feature *i* and *j*,

$\overset{―}{x}$ is the mean of this attribute,

$w_{ij}$ is the spatial weight between observations *i* and *j*,

### 3.3 Multiscale Geographically Weighted Regression (MGWR)

Initially, Geographically Weighted Regression (GWR) was considered to address spatial heterogeneity by providing localized coefficient estimates. However, due to significant multicollinearity issues identified in preliminary analyses, GWR was excluded from the final model. To address these issues, we adopted Multiscale Geographically Weighted Regression (MGWR), which enhances GWR by estimating each explanatory variable at its own optimal spatial scale (i.e., using variable-specific bandwidths). This structure helps reduce the spatial overlap of explanatory variables, thereby mitigating spatially induced multicollinearity that can arise when a single bandwidth is applied across all predictors. Additionally, since MGWR fits localized models, it can lessen multicollinearity in local subsets even when global correlations remain high [15].

Moreover, MGWR offers methodological advantages beyond addressing multicollinearity. While GWR applies a single bandwidth to all explanatory variables—implicitly assuming that all relationships operate at the same spatial scale—MGWR allows each variable to operate at its own optimal scale, offering a more realistic representation of spatial processes. This flexibility enhances model interpretability, as different factors influencing housing prices (e.g., proximity to parks vs. access to public transportation) may exert their effects over different spatial extents. Prior studies have demonstrated that MGWR improves both model fit and explanatory power compared to GWR, particularly in complex urban environments where spatial heterogeneity varies by variable [16–18]:


$$y_{i}=\sum {\mathrm{\backslash nolimits}}_{j=0}^{m}\beta_{bwj}\left(u_{i},v_{i}\right)x_{ij}+\varepsilon_{i}$$

Where:

$\beta_{bwj}\;$ is the bandwidth used in the j th location.

This approach ensures that the analysis remains robust and provides reliable insights into how urban vegetation impacts housing prices spatially and temporally.

## 4. Data and background

**Fig 1:**
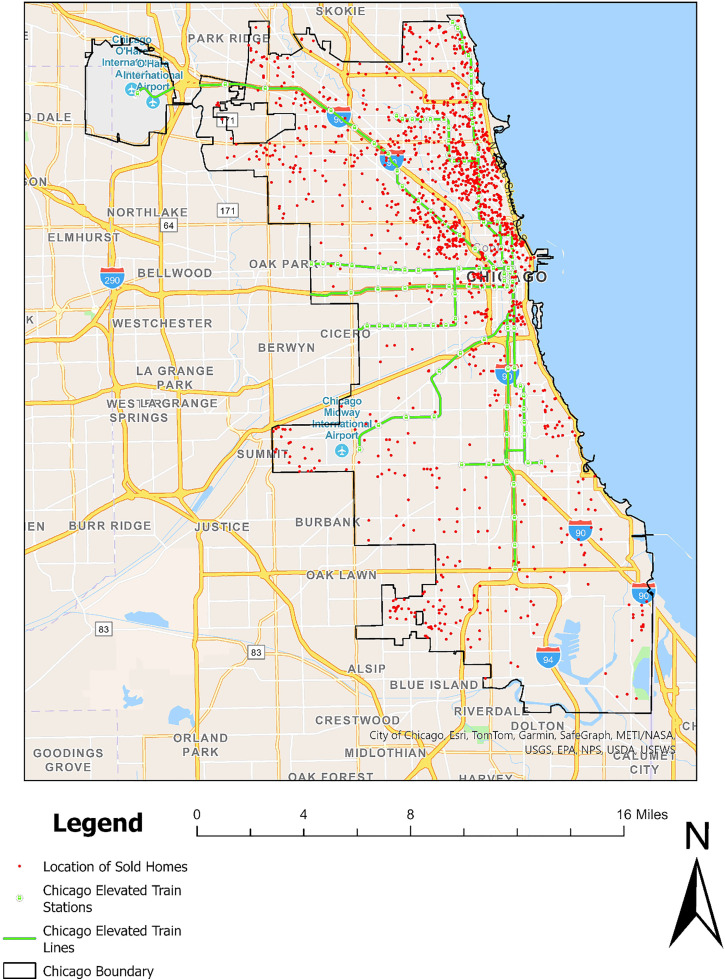
Distribution of housing transaction data in Chicago.

The scope of this study includes properties that were traded from November 2021 to November 2023, with at least one transaction occurring between 2016 and 2019, to provide a comparative analysis of pre-COVID-19 housing prices. A total of 1,381 transactions were recorded from the real estate website Zillow. The spatial distribution of these properties is depicted in (Fig 1).

**Fig 2:**
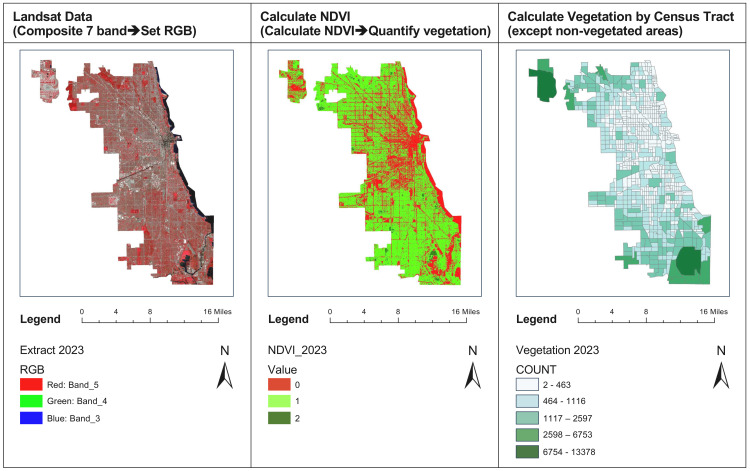
Vegetation calculation process.

**Fig 3:**
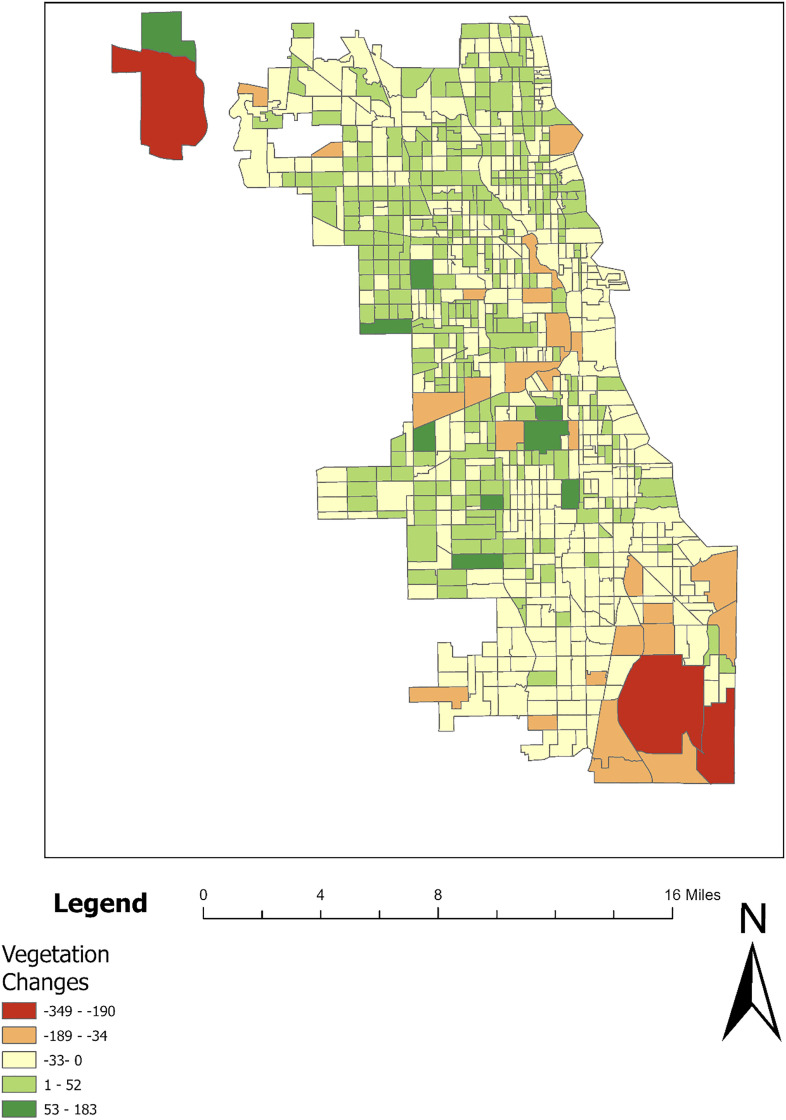
Vegetation changes in Chicago between 2019–2023.

Variables were categorized and analyzed using infrastructure (such as distance to hospitals and number of ridership) and geographic information data collected from the Chicago Data Portal, as well as Landsat data from the United States Geological Survey (USGS). For the analysis of vegetation change, the Normalized Difference Vegetation Index (NDVI) values from Landsat data for the years 2019 and 2023 were calculated using ArcGIS Pro. Only pixels with vegetation (NDVI value greater than 0.4) were considered to measure the change in vegetation count within each census tract between these years, as shown in (Fig 2) and (Fig 3). This methodology follows several studies that assess increases or decreases in vegetation at the census tract level [19–21].

In a parallel notion of utilizing high-fidelity data for accurate analysis, Chen et al. (2024) demonstrated the use of high-resolution ship imaging trajectories to enhance maritime traffic situation awareness. Their study utilized advanced imaging and tracking techniques to obtain detailed maritime data, underscoring the importance of precise data acquisition in complex scenarios [22]. This approach mirrors our use of detailed spatial data to understand the subtle impacts of urban vegetation on property values.

Beyond environmental metrics, this analysis incorporates pivotal housing characteristics such as exclusive use area, number of bedrooms, number of bathrooms, and building age. These attributes are acknowledged as fundamental drivers of housing value, directly influencing buyer preferences and market valuation [23–25].

The study further explores surrounding environmental characteristics that contribute to locational desirability and, consequently, property values. Variables such as transit ridership, distance to the nearest hospital, and ratings of primary and high schools are included to capture the indirect effects of urban accessibility and educational quality on housing markets [26–27].

Moreover, the study enriches its data set with variables reflecting the proximity to Lake Michigan, leveraging Chicago’s unique geographical feature. This inclusion not only acknowledges the aesthetic and recreational appeal of water proximity but also its proven effect in boosting housing values. Research has shown that proximity to significant bodies of water can enhance housing values due to increased desirability and recreational opportunities [28–29].

To accurately reflect the travel and accessibility within the urban fabric, all distance measurements were taken using network distances rather than Euclidean distances, aligning with best practices in spatial data analysis [30–31]. Given the heterogeneous nature of the real estate price data, a logarithmic transformation was applied to normalize distributions, simplify relationships, and stabilize variance [32–33], ensuring a robust analytical framework.

The detailed account of variables and their impact is methodically documented in Table 1 and 2, providing a comprehensive overview of the factors influencing housing prices in Chicago, accentuated by the strategic location adjacent to Lake Michigan. This comprehensive approach enhances our understanding of market dynamics, laying a solid foundation for more informed decision-making in the real estate sector.

**Table 1:** Variable types.

Variable Category	Variable Name		Value Type	Definition
**Dependent Variable**	Log Transaction Price		$(Dollar)	The logarithm of the transaction price per square foot.
**Independent Variable**	Vegetation Change		Ratio scale	Change in vegetation cover from 2019 to 2023, measured by census tract.
	COVID-19 Period		Dummy (0, 1)	Indicates the period before (0) and after (1) the COVID-19 outbreak, with the reference period being before COVID-19(ref. before COVID-19).
	Housing Characteristics	Area for exclusive use	Ratio scale	The total square footage of the target housing unit.
		Number of Bedrooms	Ratio scale	Total number of bedrooms in the housing unit.
		Number of Bathrooms	Ratio scale	Total number of bathrooms in the housing unit.
		Age of Property	Ratio scale	The difference in years between the transaction year and the year the property was built (Transaction year – Built year).
		Cooling Availability	Dummy (0, 1)	Indicates whether a cooling system is available (1) or not (0) (ref.no cooling).
		Heating Availability	Dummy (0, 1)	Indicates whether a heating system is available (1) or not (0) (ref.no heating).
		Parking Availability	Dummy (0, 1)	Indicates whether parking is available (1) or not (0) (ref.no parking).
	Surrounding Environment Characteristics	Ridership	Ratio scale	Total monthly number of passengers at the nearest station, based on the transaction month.
		Distance to Nearest Train Station	Ratio scale	Distance in miles to the nearest elevated train station.
		Primary School Rating	Ratio scale	Rating of the nearest primary/high school, based on *GreatSchools.org*’s comprehensive assessment scale. The scale includes four levels: Student Progress Rating or Academic Progress Rating, College Readiness Rating, Equity Rating, and Test Score Rating.
		High School Rating	Ratio scale	
		Distance to Nearest General Hospital	Ratio scale	Distance in miles to the nearest general hospital.
		Proximity to Waterbody	Dummy (0, 1)	Dummy variable indicating whether the property is within 1 mile (1) or exceeds 1 mile (0) from the nearest significant waterbody. (ref.exceed 1 mile).
		Crime Frequency	Ratio scale	Indicates the number of violent or serious crimes reported from 2001 to 2023 within the census tract of the property.

**Table 2:** Descriptive statistics of key variables.

Variable Category	Variable Name		Unit	Minimum	Maximum	Mean	Standard Deviation	Median
**Dependent Variable**	Log Transaction Price		$(USD)	4.116	7.262	5.656	0.445	5.724
**Independent Variable**	Vegetation Change		Ratio scale	−183	190	2.772	20.355	2
	COVID-19 Period		Dummy (0, 1)	0	1	0.5	0.5	0.5
	Housing Characteristics	Area for exclusive use	Ratio scale	459	6,100	1,695.78	841.558	1,500
		Number of Bedrooms	Ratio scale	0	9	2.772	1.233	3
		Number of Bathrooms	Ratio scale	0	8	2.254	1.025	2
		Age of Property	Ratio scale	0	151	55.773	41.686	53
		Cooling Availability	Dummy (0, 1)	0	1	0.871	0.335	1
		Heating Availability	Dummy (0, 1)	0	1	0.894	0.308	1
		Parking Availability	Dummy (0, 1)	0	1	0.613	0.487	1
	Surrounding Environment Characteristics	Ridership	Ratio scale	0	441,035	103,422.067	82,869.421	78,868.5
		Distance to Nearest Train Station	Ratio scale	0.023	8.476	1.113	1.274	0.607
		Primary School Rating	Ratio scale	1	10	5.356	2.360	5
		High School Rating	Ratio scale	1	10	3.074	2.396	2
		Distance to Nearest General Hospital	Ratio scale	0.007	7.319	1.435	1.114	1.155
		Proximity to Waterbody	Dummy (0, 1)	0	1	0.820	0.384	1
		Crime Frequency	Ratio scale	21	2,497	338.982	318.972	222

**Fig 4:**
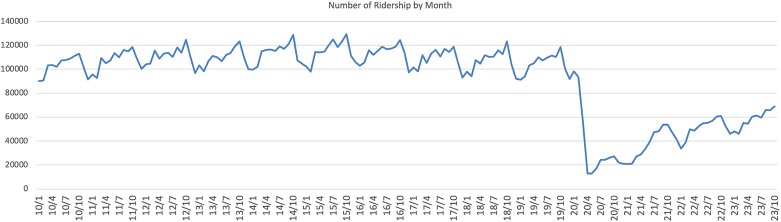
Monthly ridership trends in Chicago elevated train.

The decision to segment the data into pre- and post-COVID periods is based on the timeline of the pandemic. COVID-19 was first reported in Wuhan, China, in December 2019, with the second case in the United States identified in Chicago on January 24, 2020, following the first case in Washington (Centers for Disease Control and Prevention). Moreover, a significant drop in the number of passengers on the Chicago Elevated Train since January 2020, as shown in (Fig 4), highlights the effects of the pandemic. Consequently, for the purposes of this analysis, the time before 2019 is categorized as pre-COVID, without the pandemic's influence, and the period from 2020 onward is considered the COVID-impacted period.

## 5. Empirical results

Our empirical analysis provides significant insights into the influence of urban vegetation on housing prices, delineating effects both before and after the COVID-19 pandemic. We utilized Ordinary Least Squares (OLS) and Multiscale Geographically Weighted Regression (MGWR) models to unpack these dynamics.

### 5.1 Ordinary Least Squares (OLS) analysis

The OLS analysis presented in Table 3 reveals significant effects for nearly all variables, with the exception of high school ratings. The ridership variable demonstrates a minimal coefficient, indicating a negligible impact on housing prices. The model accounts for approximately 48% of the variance, which is indicative of a good fit. All VIF values remain below 5, thereby confirming the absence of multicollinearity concerns.

**Table 3:** Ordinary least square analysis result.

Variable Name		Ordinary Least Squares (OLS)		
**B**	**Std Error**	**VIF**
**(Intercept)**		5.596185	0.044461	
**Vegetation Change**		0.001366^***^	0.000325	1.115154
**COVID-19 Period**		−0.112247^***^	0.015354	1.358778
**Housing Characteristics**	Area for exclusive use	−0.000129^***^	0.000015	3.667977
	Number of Bedrooms	−0.022254^**^	0.008400	2.446526
	Number of Bathrooms	0.107112^***^	0.012031	3.483014
	Age of Property	−0.002029^***^	0.000175	1.282588
	Cooling Availability	0.263373^***^	0.025024	1.525951
	Heating Availability	−0.111098^***^	0.027647	1.554257
	Parking Availability	0.054478^***^	0.015728	1.326772
**Surrounding Environment Characteristics**	Ridership	0.000001^***^	0.000000	1.421648
	Distance to Nearest Train Station	−0.145654^***^	0.007207	1.484469
	Primary School Rating	0.037895^***^	0.003113	1.256170
	High School Rating	0.005323	0.003255	1.402766
	Distance to Nearest General Hospital	−0.016489^*^	0.006849	1.197882
	Proximity to Waterbody	0.051588^**^	0.019698	1.231590
	Crime Frequency	−0.000132^***^	0.000022	1.226467
**Adjusted** $R^{2}$		0.483499		

Dependent variable: Transaction price per square foot (USD) -

* p < 0.05,

** p < 0.01,

*** p < 0.001.

Our results demonstrate that housing prices are positively associated with increased vegetation, the pre-COVID period, smaller housing areas, fewer bedrooms, more bathrooms, newer constructions, cooling systems, and available parking. Closer proximity to transit stations, higher scores of nearby primary schools, hospitals, and water bodies, along with lower crime rates, are also associated with higher housing prices. These findings align with previous research, underscoring the enduring value of accessibility and quality infrastructure in urban real estate markets.

In the context of Chicago, housing prices are notably higher in the downtown area, where properties are typically smaller and possess fewer rooms compared to those in the suburbs. This pattern supports the observed trend that smaller exclusive areas and fewer bedrooms are associated with higher housing prices, a plausible outcome given the premium on central urban locations. However, the observation that fewer heating systems correlate with higher property values deviates from prior studies.

### 5.2 Spatial autocorrelation

The outcomes of the Moran’s I index measurement are presented in Table 4. Specifically, the Moran’s I index yields a positive value of 0.895803, which confirms a strong spatial correlation among housing prices within Chicago. This result underscores the importance of considering spatial dependency in urban economics analyses as ignoring such correlations can lead to biased estimates of effects. The high Moran’s I index signals that prices in nearby locations are more similar than would be expected under random distribution, justifying the subsequent application of geographically weighted regression techniques to discern local influences that might be obscured in global models.

**Table 4:** Global Moran’s I summary.

Moran’s Index	0.895803
**Expected Index**	−0.000414
**Variance**	0.000699
**Z-Score**	33.886036
**P-Value**	0.000000

### 5.3 Multiscale Geographically Weighted Regression (MGWR)

The MGWR model enhances our understanding of the spatial dynamics influencing housing prices. Table 5 compares the performance metrics of the OLS and MGWR models. Although the AICc for the MGWR model is higher than for OLS, suggesting a less parsimonious model, this does not necessarily indicate inferior performance in the context of spatial analysis.

**Table 5:** Comparison of OLS and MGWR model indicator.

	$R^{2}$	Adjust$R^{2}$	AICc	Residual
**OLS**	0.4869	0.4835	1422.5811	250.9959
**MGWR**	0.9298	0.8979	2631.8876	169.5713

A key advantage of MGWR is its ability to model spatial heterogeneity, allowing for the exploration of how relationships between variables vary across different geographic locations. This capability is particularly crucial in urban studies, where the influence of factors such as vegetation, infrastructure, and demographic characteristics can differ significantly from one neighborhood to another.

Despite the higher AICc, the MGWR model's substantially higher adjusted $R^{2}$ and lower sum of squared residuals indicate that it provides a better fit to the data. The adjusted $R^{2}$ of 0.8979 for MGWR, compared to 0.4835 for OLS, reveals that MGWR explains a much larger proportion of the variance in housing prices. This suggests that the local variations captured by MGWR are crucial for understanding the spatial patterns that a global model like OLS might overlook.

Furthermore, while OLS assumes a constant relationship across space, MGWR adapts to local data conditions, potentially capturing complex interactions that are masked in a global analysis. This makes MGWR particularly useful in real estate economics, where local context and micro-location factors can dramatically influence property values.

Therefore, while the OLS model may appear more parsimonious, MGWR’s ability to address spatial heterogeneity and provide a more detailed and contextually relevant analysis makes it a compelling choice for our study objectives. The detailed results of MGWR, as shown in Table 6, indicate that the impacts of vegetation changes on housing prices vary substantially across different neighborhoods, contrary to initial expectations. A decrease in greenery is paradoxically associated with an increase in housing prices in some areas, suggesting complex underlying urban dynamics that influence housing values. These detailed findings underscore the necessity of localized analysis in understanding the multifaceted nature of urban real estate markets.

**Table 6:** Multiscale Geographically Weighted Regression (MGWR) result.

Variable Name		Multiscale Geographically Weighted Regression (MGWR)						
		Mean	Standard Deviation	Min	Median	Max	Bandwidth (%of Features)	Significance (%of Features)
**(Intercept)**		−0.0711	0.6184	−1.9318	0.0494	1.2388	30 (1.24)	718 (29.74)
**Vegetation Change**		−0.0331	0.0188	−0.1059	−0.0297	−0.0118	1,421 (58.86)	672 (27.84)
**CCOVID-19 Period**		−0.1722	0.0053	−0.1875	−0.1711	−0.1658	2,066 (85.58)	2,414 (100.00)
**Housing Characteristics**	Area for exclusive use	−0.3027	0.2909	−1.5055	−0.2369	0.2852	72 (2.98)	1,462 (60.56)
	Number of Bedrooms	0.1161	0.2838	−0.8880	0.1185	1.2096	30 (1.24)	396 (16.40)
	Number of Bathrooms	0.1473	0.1923	−0.4704	0.1564	0.6955	88 (3.65)	882 (36.54)
	Age of Property	−0.1148	0.2647	−0.9169	−0.0797	0.7844	30 (1.24)	474 (19.64)
	Cooling Availability	0.0895	0.1687	−0.5573	0.0756	0.6000	50 (2.07)	488 (20.22)
	Heating Availability	−0.0523	0.0116	−0.0645	−0.0567	−0.0221	2,035 (84.30)	2,246 (93.04)
	Parking Availability	0.0384	0.0285	−0.0151	0.0325	0.1059	726 (30.07)	746 (30.90)
**Surrounding Environment Characteristics**	Ridership	0.0188	0.0409	−0.0731	0.0154	0.1058	511 (21.17)	466 (19.30)
	Distance to Nearest Train Station	−0.1825	0.0029	−0.1858	−0.1836	−0.1739	2,414 (100.00)	2,414 (100.00)
	Primary School Rating	0.0947	0.0150	0.0563	0.0988	0.1123	1,156 (47.89)	2,414 (100.00)
	High School Rating	0.1190	0.0015	0.1135	0.1195	0.1203	2,414 (100.00)	2,414 (100.00)
	Distance to Nearest General Hospital	0.0225	0.2060	−0.6841	0.0067	1.1942	30 (1.24)	142 (5.88)
	Proximity to Waterbody	0.0288	0.0007	0.0276	0.0286	0.0309	2,414 (100.00)	396 (16.40)
	Crime Frequency	−0.0908	0.0001	−0.0912	−0.0907	−0.0906	2,414 (100.00)	2,414 (100.00)

Table 6 elucidates the varied influences of different variables on the logarithm of transaction prices per square foot of real estate, revealing intricate dynamics within the housing market. The intercept's negative mean value suggests generally lower price levels once adjusted for all variables. The negative coefficient for the COVID-19 Period indicates that housing prices were notably higher before the pandemic, with this factor exerting a substantial influence across all regions (100% significance).

In contrast, the coefficient for Vegetation Change is negative on average, implying that increases in vegetation cover from 2019 to 2023 were associated with lower housing prices. However, this effect was not uniformly significant across all regions. While 58.86% of the study areas were affected by vegetation change, only 27.84% exhibited a statistically significant relationship. This suggests that vegetation cover changes played a role in shaping housing prices, but their impact varied across different locations.

Housing characteristics exhibit mixed effects, with many variables showing limited statistical significance. Properties designated for exclusive use and those without heating systems correlate with higher housing prices, as reflected by their negative coefficients. Similarly, the negative coefficient for the age of property highlights that newer properties tend to command higher prices, though this trend is only statistically significant in 19.64% of the analyzed areas.

Conversely, positive coefficients for the number of bedrooms and bathrooms suggest that properties with more rooms tend to command higher prices. The presence of a cooling system, as indicated by a positive coefficient for Cooling Availability, enhances property values. Similarly, Parking Availability positively impacts property prices, but its effect is statistically significant in only 30.90% of the data set. This suggests that while parking availability may be perceived as a valuable amenity, its actual impact on housing prices is not consistently strong across all areas.

Compared to housing characteristics, surrounding environmental factors exhibit stronger and more consistent influences on property values, as they generally have higher statistical significance across all analyzed areas. Distance to the nearest train station, school ratings, and crime frequency show statistically significant relationships across all study areas, demonstrating their strong influence on property values. The negative coefficient for Distance to the Nearest Train Station confirms that properties closer to train stations are consistently valued higher across all analyzed areas.

However, Ridership at the nearest train station does not show a consistently strong impact on housing prices, as it is statistically significant in only 19.30% of the data set. Proximity to hospitals was only significant at 5.88%. Proximity to water bodies within a mile also contributes positively to property values, but its significance is limited to 16.40% of the analyzed areas.

**Fig 5:**
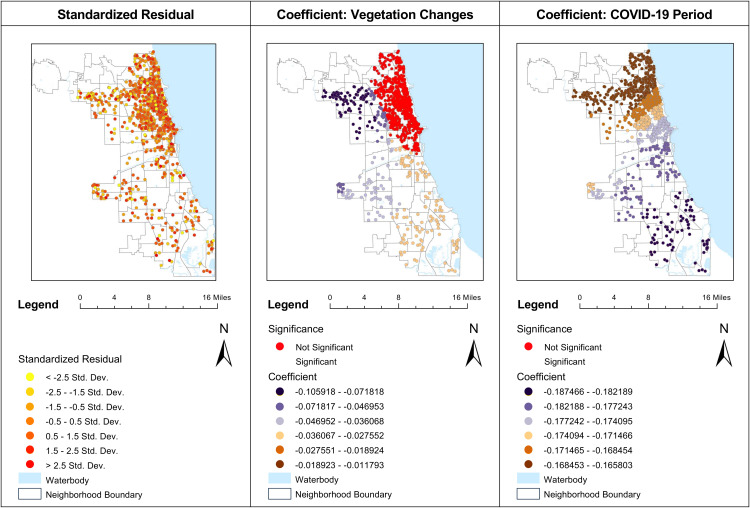
MGWR analysis of standardized residuals, vegetation changes, and COVID-19 factors.

**Fig 6:**
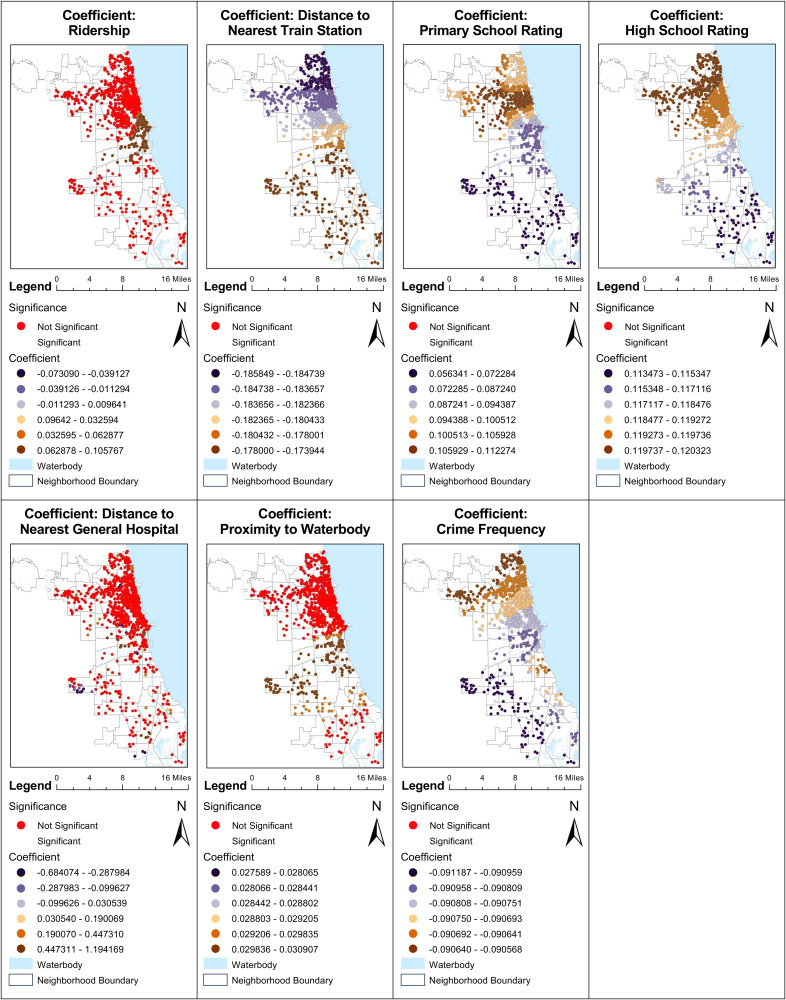
MGWR analysis of surrounding environmental characteristics.

**Fig 7:**
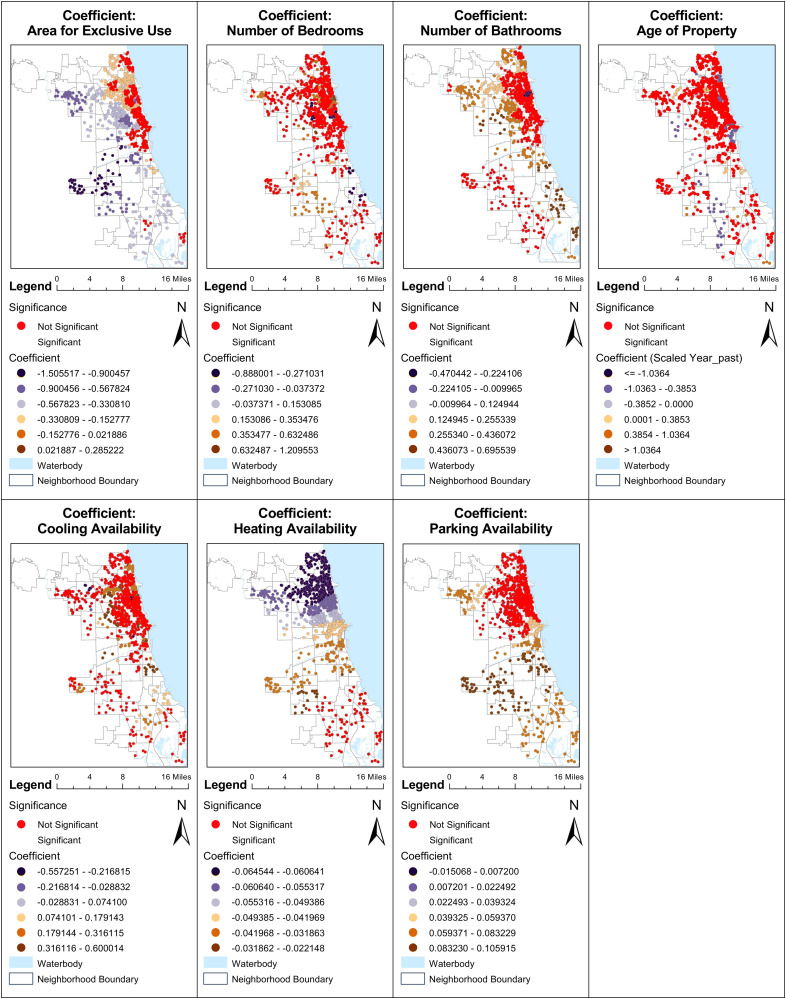
MGWR analysis of housing characteristics.

Below (Fig 5 , 6 and 7) is a map visualizing these dynamics, providing a spatial representation of how various factors influence housing prices across different neighborhoods.

Upon examining the standardized residuals in (Fig 5), we observe a broad distribution. The red dots, representing standardized residuals greater than 2.5, signify areas where the MGWR model overestimates property prices. Conversely, yellow dots, indicating residuals less than −2.5, pinpoint locations where prices are significantly underestimated. A key observation is the variability in the impact of vegetation change across different parts of Chicago (Fig 5). Notably, in the northern riverine areas, vegetation changes had no significant effect on house prices, whereas in the northwestern and southern regions, there was a strong negative correlation between increased vegetation and lower house prices.

The COVID-19 period (Fig 5) and crime frequency (Fig 6) metrics reveal a similar pattern. Data across the board is significant, with the southern areas exhibiting greater sensitivity to the pre-COVID-19 period and lower crime rates, thus correlating with higher property values. Conversely, the northern areas, shown in dark brown, exhibit a slightly less negative coefficient, suggesting a lower impact of COVID-19 and crime frequency on housing prices in these locales.

The analysis also underscores the significant role of education quality in property valuation (Fig 6). Higher ratings for primary and high schools consistently correlate with increased property values, a trend that is more pronounced in the northern parts of Chicago. This indicates that premium education facilities substantially enhance property values in these areas. However, in zones marked in dark blue, the influence of school ratings on property prices is not as significant.

Additionally, the proximity to train stations emerges as a critical factor, with closer distances correlating strongly with higher property prices, especially in the northern regions (Fig 6). This trend highlights a greater sensitivity to transit accessibility in the northern parts of the city compared to the south.

**Fig 8:**
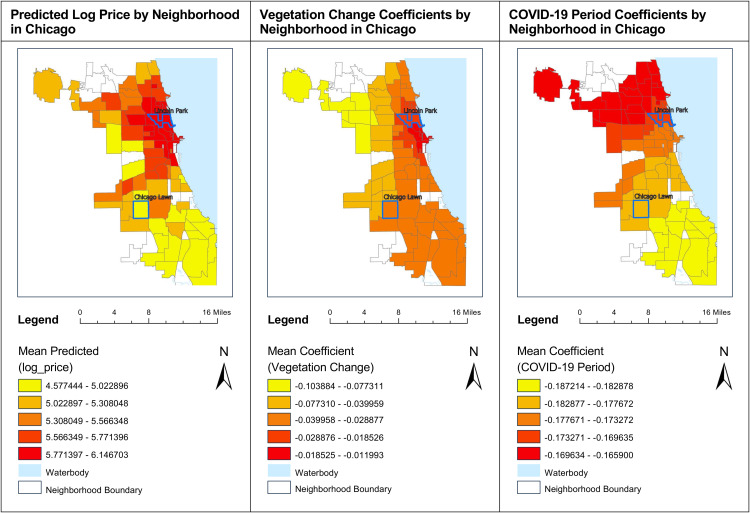
Comparative analysis of housing prices, vegetation impact, and COVID-19 effects in Lincoln Park and Chicago Lawn, Chicago.

Other factors such as the number of bedrooms, bathrooms, property age, cooling availability, parking availability, ridership, and proximity to general hospitals show varying degrees of influence, with many exhibiting insignificant results across different regions. This varied impact calls for a thorough, detailed regional analysis to fully understand the spatial dynamics of urban vegetation and its implications on housing prices. The forthcoming (Fig 8) delves deeper into these regional differences, providing a comparative analysis of housing prices, vegetation impact, and COVID-19 effects in specific neighborhoods such as Lincoln Park and Chicago Lawn.

(Fig 8) presents a comparative analysis of predicted log prices, vegetation change coefficients, and COVID-19 period coefficients across neighborhoods in Chicago, with a particular focus on Lincoln Park and Chicago Lawn.

The first map highlights predicted log prices, where deeper red shades signify higher log prices. This vividly illustrates Lincoln Park as a high-value housing market compared to Chicago Lawn, which is represented in lighter shades, indicating significantly lower housing prices.

The second map shows the coefficients for vegetation changes. In this map, lighter colors correspond to larger negative coefficients, while darker shades indicate smaller negative impacts. Lincoln Park consistently exhibits darker shades, suggesting that vegetation changes have had a relatively mild negative influence—or possibly no significant impact—on housing prices in this area, as previously observed in (Fig 5) where most vegetation change coefficients in Lincoln Park were statistically insignificant. In contrast, Chicago Lawn is represented by lighter colors, indicating a more pronounced negative correlation between vegetation changes and housing prices, signifying the neighborhood's greater vulnerability to vegetation changes.

The third map illustrates the coefficients for the COVID-19 period. Here, Lincoln Park again displays darker shades compared to Chicago Lawn, reflecting a smaller negative impact of the pandemic on housing prices. In contrast, Chicago Lawn’s lighter colors indicate that the COVID-19 period had a more significant adverse effect on its housing market.

This comparative visualization underscores the varying degrees of resilience across neighborhoods in response to external factors such as environmental changes and global health crises. Lincoln Park demonstrates remarkable stability in housing values despite these challenges, attributable to its socioeconomic advantages and established market conditions. On the other hand, Chicago Lawn appears more susceptible to both vegetation changes and the COVID-19 pandemic, highlighting the complex interplay of geographic, environmental, and socioeconomic factors that shape real estate dynamics in Chicago.

## 6. Discussion

This study extends our understanding of the relationship between urban vegetation and housing prices in Chicago, employing OLS and MGWR models to underscore the spatial variability of these effects across different neighborhoods in the post-COVID-19 era. While foundational research by Anderson and Cordell (1988) and Tyrväinen and Miettinen (2000) has established a positive correlation between green spaces and property values due to aesthetic and recreational benefits [1–2], our findings reveal a more complex landscape. The impact of green spaces is not uniformly positive and varies significantly by neighborhood, reflecting broader socioeconomic disparities.

As numerous studies have indicated, income levels in Chicago are higher in the north than in the south, which also points to pronounced social inequalities [34–37]. These socioeconomic disparities manifest in differing sensitivities to changes in urban vegetation and environmental conditions, with lower-income areas in the south showing greater vulnerability to these changes. This differential impact underscores the need for targeted urban planning and policy interventions that address these disparities effectively.

Interestingly, while factors related to the built environment and accessibility, such as proximity to amenities and infrastructure, have a pronounced influence on housing prices, our study uncovers how these influences differ in low-income and high-income areas. In regions like the north of Chicago, where income levels are higher, school ratings and access to public transportation appear to be more significant determinants of housing values. In contrast, in the south, which generally has lower income levels, changes in vegetation and the environment before and after COVID-19 have a more substantial impact on housing prices. This suggests that lower-income areas may be more vulnerable to environmental changes than previously thought.

Additionally, the analysis of accessibility to transportation hubs reveals that it is not solely the income differences driving this disparity but also the actual availability of developed stations. (Fig 1) illustrates that the Chicago Elevated Train network is denser in the north than in the south, providing a structural advantage to these neighborhoods that goes beyond mere socioeconomic status.

Our localized analysis using MGWR shows that preferences for urban features are intricately tied to specific urban contexts and are not uniform across all neighborhoods. The detailed insights provided by the MGWR model in this study highlight the significant spatial heterogeneity in how urban vegetation impacts property values. This level of detail is crucial, as it emphasizes the importance of considering both socioeconomic and infrastructural factors when planning urban development.

Our results do not uniformly support the economic benefits of green spaces; instead, they illustrate that while green spaces can enhance property values, their impact is significantly mediated by more impactful urban factors such as local socio-economic conditions and broader urban policy initiatives. This suggests that the integration of green spaces within comprehensive urban development plans is crucial for maximizing their benefits and ensuring equity across communities.

For a more resilient and sustainable post-pandemic urban development, our findings advocate for an integrated approach where urban planning not only considers the aesthetic and recreational benefits of green spaces but also actively incorporates these elements into broader urban development policies aimed at mitigating socio-economic disparities and enhancing community resilience.

## 7. Conclusion

This study has provided detailed insights into the relationship between urban vegetation and housing prices in Chicago using OLS and MGWR models, revealing that the impact of green spaces on property values is influenced by several socio-economic and environmental factors and varies significantly across different neighborhoods.

Our findings challenge the simplistic assumption that urban green spaces invariably enhance housing values. Instead, we observe that the impact of green spaces on property values is highly localized and varies significantly across different neighborhoods. In some areas, increases in vegetation were actually associated with decreases in housing prices, suggesting that the economic benefits of green spaces are heavily mediated by other local factors.

Furthermore, the COVID-19 pandemic has reshaped urban development patterns and housing market preferences, emphasizing the need for urban planning that can accommodate rapid shifts in societal behaviors and preferences. The findings suggest that while green spaces are valued less during such disruptions, their benefits are not uniformly distributed across different socio-economic groups, highlighting the role of urban planning in managing disparities.

However, the study acknowledges certain limitations that may affect the interpretation of the findings. Firstly, the analysis did not account for the reason why housing prices are higher in houses without heating facilities, which remains an area for further investigation. Secondly, while the study utilized NDVI measurements to assess green spaces, this method may not capture the varied value of different types of greenery. As noted by Yan et al. (2023), measures such as eye-level greenness provide a more comprehensive understanding of how green spaces are perceived and used by individuals, suggesting that future research might benefit from incorporating such measures to better assess the impact of urban greenery on housing prices [38].

Based on the findings, it is recommended that urban green space planning and implementation be integrated with broader urban policy initiatives to enhance their effectiveness and equity across communities. Tailoring green space interventions to the specific dynamics and needs of each neighborhood could help mitigate socio-economic disparities and enhance the resilience of urban areas against future socio-economic shocks.

In conclusion, this study contributes to the growing body of literature on the economic valuation of urban green spaces by providing a detailed analysis of how their benefits vary spatially within a city. It highlights the importance of incorporating diverse and detailed measures of greenness in urban economic research and the critical role of inclusive and thoughtful urban design and planning in creating sustainable, resilient, and livable urban environments for all residents.

## Supporting information

S1 Data Raw data of housing transactions and vegetation indices used in the analysis. (XLSX)
